# Supplementary material for: Heterozygous and Homozygous Variants in SORL1 Gene in Alzheimer’s Disease Patients: Clinical, Neuroimaging and Neuropathological Findings
Source: Int J Mol Sci. 2022 Apr 11;23(8):4230. doi: 10.3390/ijms23084230 (PMC9024679; doi:10.3390/ijms23084230)
Supplement: Supplementary file 1 [file ijms-23-04230-s001.zip › ijms-1660070-supplementary.pdf]

Supplementary Table S1. Candidate variants identified in neurodegenerative dementia causal genes

Patient ID	Gene	Transcript ID	cDNA	Protein change	ACMG classification[¥]	OMIM Disease	ApoE genotype
**19NG1297**	*NOTCH3*	NM_000435.2	c.1819C>T	p.R607C	Pathogenic	CADASIL[*]	3/3
**20NG0521**	*MAPT*	NM_001123066	c.2194C>T	p.P732S	Likely Pathogenic	Frontotemporal dementia	3/3
**19NG0814**	*PSEN1*	NM_000021.3	c.313T>G	p.F105V	Likely Pathogenic	Alzheimer's disease	3/3
**20NG0518**	*APP*	NM_000484.3	c.539G>T	p.R180L	VUS	Alzheimer's disease	3/3
**18NG0103**	*APP*	NM_000484.3	c.982C>T	p.R328W	VUS	Alzheimer's disease	3/3
**19NG0871**	*SQSTM1*	NM_003900.4	c.436_462dup	p.P146_C154dup	VUS	Frontotemporal dementia	3/3
**19NG1113**	*NOTCH3*	NM_000435.2	c.1931T>A	p.V644D	VUS	CADASIL[*]	3/3

[*] Cerebral arteriopathy with subcortical infarcts and leukoencephalopathy
[¥] VUS: variant of unknown significance
